# Anemia and outcomes in cardiac surgery

**DOI:** 10.1016/j.bjane.2024.844504

**Published:** 2024-04-25

**Authors:** Luiz Guilherme V. da Costa, Fabio V. Papa, Gregory M.T. Hare, Marcello F. Salgado-Filho, Eric B. Lineburger, André P. Schmidt

**Affiliations:** a Hospital Israelita Albert Einstein, Departamento de Anestesiologia, São Paulo, SP, Brazil; b University of Toronto, St. Michael's Hospital, Department of Anaesthesia, Toronto, Canada; c Hospital São José, Departamento de Anestesia e Tratamento da Dor, Criciúma, SC, Brazil; d Hospital São José, Centro de Pesquisa, Criciúma, SC, Brazil; e Universidade do Extremo Sul Catarinense, Criciúma, SC, Brazil; f Hospital de Clínicas de Porto Alegre (HCPA), Serviço de Anestesia e Medicina Perioperatória, Porto Alegre, RS, Brazil; g Santa Casa de Porto Alegre, Serviço de Anestesia, Porto Alegre, RS, Brazil; h Hospital Nossa Senhora da Conceição, Serviço de Anestesia, Porto Alegre, RS, Brazil; i Universidade Federal do Rio Grande do Sul (UFRGS), Programa de Pós-graduação em Ciências Pneumológicas, Porto Alegre, RS, Brazil; j Universidade Federal do Rio Grande do Sul (UFRGS), Programa de Pós-graduação em Ciências Cirúrgicas, Porto Alegre, RS, Brazil; k Programa de Pós-Graduação em Anestesiologia, Ciências Cirúrgicas e Medicina Perioperatória, Faculdade de Medicina da Universidade de São Paulo (FMUSP), São Paulo, Brazil.

In patients eligible for cardiac surgery, the prevalence of anemia ranges from 16% to 54%, with severe cases (hemoglobin < 10 g.dL^−1^) affecting 5.5% of individuals. Perioperative anemia significantly elevates the risk of morbidity, mortality, and organic dysfunction.^1^ Over recent decades, numerous studies have emerged to explore optimal blood transfusion strategies, determine the most effective transfusion triggers, and establish safe hemoglobin thresholds across diverse patient population.^2^^,^^3^

One of the most relevant studies in this context was the TRICC Trial,^4^ a randomized clinical trial (RCT) involving 838 euvolemic patients. The study encompassed a diverse population with hemoglobin levels < 9 g.dL^−1^ within 72 hours of admission to the critical care unit. This groundbreaking work challenged the liberal transfusion logic in force at the time, comparing a restrictive transfusion trigger arm (hemoglobin < 7 g.dL^−1^) to a liberal arm (hemoglobin < 10 g.dL^−1^). The incidences of myocardial infarction and acute pulmonary edema were statistically lower in the restrictive group. The study concluded that a more restrictive transfusion strategy proved to be at least as effective, if not superior, to the liberal strategy in critically ill patients, except for mortality in the subgroup of patients with cardiovascular disease.

In 2016, Carson et al^3^ published guidelines that revisited 31 RCTs, advocating for more restrictive transfusion trigger recommendations with hemoglobin levels < 7 g.dL^−1^ for the general population and 8 g.dL^−1^ for orthopedic patients, those undergoing cardiac surgery, and individuals with cardiovascular diseases.^5^ However, the guidelines did not include patients with acute coronary syndromes and emphasized the need for additional research concerning patients with acute myocardial infarction.

In 2018, a systematic review comprising 37 RCTs investigated mortality as the primary outcome among individuals undergoing cardiac surgery.^6^ This study concluded that hemoglobin levels between 7 and 8 g.dL^−1^ served as a safe transfusion trigger for the evaluated population compared to more liberal triggers. This approach also led to a reduction in the transfusion rate by 24%.

In 2018, another important noninferiority RCT (TRICS-III)^7^ was published. This study compared a restrictive transfusion trigger (hemoglobin < 7.5 g.dL^−1^) with a liberal trigger (hemoglobin < 9.5 g.dL^−1^ in the ICU or < 8.5 g.dL^−1^ in the ward) in patients undergoing cardiac surgery, particularly those with moderate and high risk of death. The primary outcome analyzed was a composite of overall mortality, acute myocardial infarction, stroke, or kidney disease requiring new-onset replacement therapy within six months post-surgery. The authors concluded that the restrictive strategy was noninferior to the liberal strategy group concerning the primary outcome.

In 2021, Ducrocq et al^8^ conducted a noninferiority RCT in France and Spain, which was open-label and involved patients with acute myocardial infarction and hemoglobin levels ranging between 7 and 10 g.dL^−1^. Patients were divided into two arms: the restrictive group had a transfusion trigger of hemoglobin < 8 g.dL^−1^ with a target between 8 and 11 g.dL^−1^, while the liberal group had a transfusion trigger of hemoglobin < 10 g.dL^−1^ with a target above 11 g.dL^−1^. The main objective of the study was a composite outcome of major cardiovascular complications (MACE), including death, stroke, recurrent acute myocardial infarction, or emergency revascularization. Once again, this study demonstrated the noninferiority of the restrictive group compared to the liberal group. However, an important limitation of this study was the wide lower margin of noninferiority, which could potentially encompass actual harm to patients. Additionally, the study had limited power to detect the superiority of one strategy over the other.

A recent meta-analysis from 2023,^9^ which encompassed RCTs^10^, ^11^, ^12^ enrolling patients with acute coronary syndrome, did not reveal a statistically significant difference between the liberal and restrictive strategies concerning outcomes such as mortality, acute myocardial infarction, revascularization, and the composite outcome of these events.

Similarly, in 2023, the Red Blood Cell Transfusion 2023 AABB International Guidelines^13^ were published, reviewing 45 RCTs. These guidelines recommended a hemoglobin trigger of 7 g.dL^−1^ for hospitalized and stable patients, 7.5 g.dL^−1^ for patients undergoing cardiac surgery, and 8 g.dL^−1^ for orthopedic patients and those with previous cardiovascular comorbidities. However, there was a low level of evidence for transfusion recommendations in patients with acute myocardial infarction.

To address this issue, the MINT (Myocardial Ischemia and Transfusion) trial^14^ was designed. This multicenter study aimed to shed light on the matter by enrolling patients with acute myocardial infarction. Participants were divided into two arms: one with a transfusion cutoff strategy of 7–8 g.dL^−1^ of hemoglobin, and another more liberal arm with a hemoglobin cutoff < 10 g.dL^−1^. The primary outcome was a composite of recurrent acute myocardial infarction and death. The study found no statistical difference between the two arms. However, the authors expressed uncertainty about ruling out the harmful effects of the restrictive strategy. Subsequently, a Bayesian analysis was published, demonstrating the potential harm associated with the restrictive strategy in this population.^3^

Perioperative anemia may impact outcomes concerning multiple target organs, including the heart, kidneys, and central nervous system. Karkouti et al^2^ addressed the association between anemia and the development of renal dysfunction, underscoring the significance of preoperative hemoglobin optimization in mitigating high mortality rates in cardiac surgery. Furthermore, red blood cell transfusions seem to be implicated in this outcome, attributed to factors such as the reduction in levels of 2,3-DPG, pro-inflammatory effects, leukocyte activation, and coagulation activation.^2^

Interestingly, in this issue of the *Brazilian Journal of Anesthesiology*, Sari et al^15^ published an innovative retrospective subanalysis of the DECADE trial.^16^ This study revealed no statistically significant association between various hemoglobin levels and delirium or atrial fibrillation in patients undergoing cardiac surgery. This conclusion was further supported by a robust Cox regression analysis, which was adjusted for clinically relevant variables.

Notably, there is an ongoing international multicenter randomized controlled trial focusing on transfusion thresholds in younger cardiac surgery patients. Known as TRICS-IV,^17^ this open-label randomized controlled trial compares two commonly employed transfusion strategies in moderate to high-risk patients aged 65 years or younger who are undergoing cardiac surgery with cardiopulmonary bypass. It employs a superiority trial design and aims to enroll approximately 1,440 patients. The study tests the hypothesis that liberal transfusion is superior for younger patients undergoing cardiac surgery, based on previous evidence generated by subgroup analyses from the TRICS III trial.^7^ The primary hypothesis suggests that maintaining a higher hemoglobin concentration for red blood cell transfusion (liberal transfusion strategy) will yield better outcomes compared to a restrictive strategy in terms of vital organ function (specifically the heart, brain, and kidneys) and lower mortality rates six months post-cardiac surgery. Hence, this study holds the potential to provide valuable new evidence in this field.

In summary, understanding key aspects of perioperative anemia is essential to guide professionals in making transfusion decisions in cardiac surgery. This entails considering the balance between risks and benefits, as well as the associated costs, with the ultimate goal of mitigating morbidity and mortality in this vulnerable population.

## Conflicts of interest

The authors declare no conflicts of interest.
